# How Austria Deals with Drunkards

**Published:** 1896-06

**Authors:** 


### How Austria Deals with Drunkards.

Austria proposes to deal with persistent drunkards by treating them as mentally incapable, and detaining them in special retreats for a term of two years. They may go in of their own accord or on compulsion, but can not leave at will until their term has expired, except in certain cases on probation. Persons may be sent to the retreat either by order of the magistrate or on the petition of the parents or children, or of the husband and wife, or trustees, or of the chief of a lunatic asylum in which a drunkard may be detained. Inebriates may further be assigned to retreats by the action of the public prosecutor, or by the mayor of the town or village in which the habitual drunkard resides. In all cases the inebriate must be legally tried and convicted, the court being bound to hear witnesses, including the drunkard himself, as well as the doctors, more especially experts on mental diseases. The term of detention will be generally for two years, but the patient may be released on leave after one year, but will be confined again in case he relapses into his former bad habits.—*Albany Med. Annals.*

